# Editorial: Impact of SARS-CoV-2 on Antibiotic Resistance: Evolution of Treatment and Control Strategies

**DOI:** 10.3389/fmolb.2023.1238479

**Published:** 2023-08-02

**Authors:** Sanket Kaushik, Vijay Kumar Srivastava, Biswajit Mishra

**Affiliations:** ^1^ Amity Institute of Biotechnology, Amity University Rajasthan, Jaipur, India; ^2^ Division of Infectious Diseases, The Miriam Hospital, Alpert Medical School of Brown University, Providence, RI, United States

**Keywords:** antibiotic resistance, SARC V2, pandemic (COVID-19), bacterial pathogen, MDR—multi drug resistant

The unselective and high usage of antibiotics was reported during the COVID-19 pandemic in several cases, due to which resistant hospital-onset infections, and deaths were increased (Bara et al., 2022; Rizvi and Ahammad, 2022; Tanne, 2022). The use of antibiotics during the pandemic was not for the treatment of COVID-19; instead, they were used because of ambiguity in the initial diagnosis of people showing symptoms of respiratory illness and having probable co-infections with bacteria (Sharifipour et al., 2020; Langford et al., 2022). However, due to this, uncertainty and unselective usage of antibiotics several antimicrobial-resistant strains could developed (Lai et al., 2021). In this regard, understanding the emergence and evolution of antimicrobial resistance in COVID-19 patients is essential.

The goal of this Research Topic was to study the global impact of COVID-19 on AMR and to integrate previous and current knowledge in the field of antibiotic resistance, right from diagnosis, pathophysiology, and novel therapeutics. However collectively, four articles were accepted for publication in the Research Topic to study how SARS-CoV-2 has changed the dynamics of anti-microbial resistance (AMR) globally.

A research article published by Gomez et al. reported the presence of multiple microorganisms as a co-infection in COVID-19 patients. In the study, patients hospitalised from January to May 2021 with a positive test for COVID-19 were identified. Other criteria were that these patients were also reported to have co-infections with more than one microorganism. For the identification of microorganisms, different samples were collected from the respiratory tract, blood, urine, etc. Results indicated the presence of 20 different microorganisms in the samples, with *Pseudomonas aeruginosa* being the most common bacterial pathogen, followed by *Klebsiella pneumoniae* and *Acinetobacter baumanii*. Overall, the study reported the presence of several bacterial pathogens in the COVID-19 scenario and also emphasised the need to minimise such bacterial co-infections during times when we do not have sufficient medical facilities.

Another research article by Pushan et al. compared the features of reported Indian H1N1 strains and the closest pandemic strain, A/California/04/2009. The paper mainly focused on the cell surface protein hemagglutinin. This protein plays a major role in the attachment of the pathogen to the host cell, leading to its entry into the host. Extensive sequence and structural analysis of the hemagglutinin protein from Indian H1N1 strains and the A/California/04/2009 strain was performed, which disclosed several point mutations in the Indian HINI hemagglutinin protein as compared to the hemagglutinin from the A/California/04/2009 strain. These mutations resulted in differences in the sequence and structure of both strains, leading to different binding of both strains to the host cell surface. It was reported that these mutations in hemagglutinin from Indian H1N1 increased its adaptability in the host.

Roy et al. targeted SARS-CoV-2 to test a few FDA-approved drugs that were reported to bind G-quadruplexes (G4s). These are higher-order DNA and RNA secondary structures formed from G-rich sequence. G4s affect important processes inside the cell as they are reported to interact with several endogenous proteins, including helicases, transcription factors, epigenetic and chromatin remodelers, etc. Therefore, they play a major role in regulating several important processes inside the cell, including the replication and transcription of the virus. In this paper, two potential G4s (pG4s) were identified to target the SARS-CoV-2 genome using FDA-approved drugs that can bind G4s: chlorpromazine (CPZ) and prochlorperazine (PCZ). Interestingly, treatment of CPZ or PCZ resulted in noteworthy changes in lung pathology and the lung viral load of SARS-CoV-2. The binding of the drugs to the pG4s was also confirmed by isothermal calorimetry; hence, the results confirmed that the pG4s can be potential targets for treatment of quickly mutating strains like SARS-CoV-2.

Finally, a review article by Kumar et al. was published that highlighted the role of specialised pro-resolving mediators (SPMs) in inducing macrophage polarisation, triggering immunological functions, speeding inflammation resolution, decreasing cytokine storms, and reducing antibiotic requirements. Viral diseases are difficult to treat due to random mutations in the viral genome; there are few targets, and hence the number of antiviral drugs is also limited. The author pointed out that the cytokine storm was the major reason for the severity of COVID-19 infection in most of the cases, and SPMs offer a promising way of treatment by controlling the severity of infection by blocking the inflammation process. The author also summed up by mentioning the importance of SPMs not only against COVID-19 but also against other viral infections where treatment options are limited.

## References

[B1] Bara W. Brun-Buisson C. Coignard B. Watier L. (2022). Outpatient antibiotic prescriptions in France: Patients and providers characteristics and impact of the COVID-19 pandemic. Antibiotics 11 (5), 643. 10.3390/antibiotics11050643 35625287 PMC9137595

[B2] Lai C. C. Chen S. Y. Ko W. C. Hsueh P. R. (2021). Increased antimicrobial resistance during the COVID-19 pandemic. Int. J. Antimicrob. agents 57 (4), 106324. 10.1016/j.ijantimicag.2021.106324 33746045 PMC7972869

[B3] Langford B. J. Soucy J. P. Leung V. So M. Kwan A. T. Portnoff J. S. (2022). Antibiotic resistance associated with the COVID-19 pandemic: A systematic review and meta-analysis. Clin. Microbiol. Infect. 29, 302–309. 10.1016/j.cmi.2022.12.006 36509377 PMC9733301

[B4] Rizvi S. G. Ahammad S. Z. (2022). COVID-19 and antimicrobial resistance: A cross-study. Sci. Total Environ. 807, 150873. 10.1016/j.scitotenv.2021.150873 34634340 PMC8500695

[B5] Sharifipour E. Shams S. Esmkhani M. Khodadadi J. Fotouhi-Ardakani R. Koohpaei A. (2020). Evaluation of bacterial co-infections of the respiratory tract in COVID-19 patients admitted to ICU. BMC Infect. Dis. 20 (1), 646–647. 10.1186/s12879-020-05374-z 32873235 PMC7461753

[B6] Tanne J. H. (2022). COVID-19: Antimicrobial resistance rose dangerously in US during pandemic, CDC says. BMJ 378, o1755. 10.1136/bmj.o1755 35835472

